# Piriformis preserving posterior approach STAR for primary and primary complex total hip arthroplasty: Excellent safety and efficacy in a single blinded prospective single surgeon cohort of 522 patients with a mean follow-up of 2 years

**DOI:** 10.1051/sicotj/2024030

**Published:** 2024-09-06

**Authors:** Eustathios Kenanidis, Vasileios F. Pegios, Eleni Tsamoura, Nikolaos Milonakis, Eleftherios Tsiridis

**Affiliations:** 1 Academic Orthopaedic Department, Aristotle University Medical School, General Hospital Papageorgiou Ring Road Efkarpia Thessaloniki 56403 Greece; 2 Centre of Orthopaedic and Regenerative Medicine (CORE), Center for Interdisciplinary Research and Innovation (CIRI)-Aristotle University of Thessaloniki (AUTH) Balkan Center, Buildings A & B, 10th km Thessaloniki-Thermi Rd P.O. Box 8318 Thessaloniki 57001 Greece; 3 Tsiridis Orthopaedic Institute – ICAROS Clinic Thessaloniki 55535 Greece

**Keywords:** STAR approach, THA, Total hip arthroplasty, Piriformis, STAR, Piriformis preservation

## Abstract

*Introduction*: STAR (Superior Transverse Anatomic Reconstruction), a piriformis-preserving posterior approach, has not been extensively studied. Our study aimed to assess the STAR approach’s safety and efficacy by recording postoperative complication rates and measuring implantation accuracy in a single surgeon prospective cohort with a mean follow-up of two years. *Methods*: The study involved 522 patients with elective primary or complex primary total hip arthroplasty (THA) performed by a senior surgeon using the STAR approach between 2019 and 2023. 63.6% of the patients were female. The mean patients’ age was 65.6 years. 19.5% of the procedures were primary complex THAs. The mean follow-up and length of stay were 2.13 years and 1.50 days. The ratio of uncemented to hybrid and standard to dual mobility liner THAs were 3:2 and 4:1. Fifty-eight patients received blood transfusions. All patients followed the same postoperative protocol. Two physicians not involved in surgery collected clinical and radiological data. Efficacy was defined as measuring the cup inclination and anteversion, stem alignment, and leg length discrepancy (LLD) using the one-month postoperative standardised supine anteroposterior pelvic X-rays. The postoperative complication rate, including dislocation and infection, defined safety. *Results*: The mean cup inclination and anteversion were 42.8^0^ (±4.9) and 19.9^0^ (±8.9), respectively. 97.5% of the stems were placed in neutral and 2.5% in varus position. The mean LLD was 3.3 ± 6.3 mm. A single deep infection was managed with two-stage revision with no recurrence, and an early traumatic dislocation in an 80-year-old woman was managed successfully with closed reduction and hip spica. Three superficial wound infections were treated with oral antibiotics. *Discussion*: The STAR approach is safe and has demonstrated excellent early-to-mid-term efficacy profile outcomes. The unobstructed acetabular and femoral intraoperative view facilitated optimal implant positioning and contributed to excellent dislocation outcomes in combination with piriformis preservation.

## Introduction

The piriformis (PF) is the most superior of the hip short external rotator (SERs) muscles [1]. During a standard posterior approach for THA, the PF tendon is resected to access the hip joint. It is usually reattached to the greater trochanter at the end of the procedure [1]. However, muscle-sparing THA techniques preserving PF have been proposed to improve hip stability and enhance postoperative recovery [1–11].

The Superior Transverse Atraumatic Anatomic Reconstruction (STAR) approach is a muscle-sparing posterior THA technique that preserves PF, iliotibial band, and quadratus femoris [1]. The first and only STAR approach study reported shorter hospital stays, lower transfusion rates, and earlier functional improvement compared to the Direct Superior Approach (DSA), a non-piriformis preserving posterior approach [1]. The authors supported that STAR provided excellent acetabular and proximal femoral exposure using any implant or technique with standard instrumentation [1]. To date, the safety and efficacy of the STAR approach have not undergone evaluation in a large cohort study.

Our primary aim was to evaluate the safety and efficacy of the STAR approach for THA. We prospectively assessed a cohort of 522 consecutive patients with end-stage hip osteoarthritis who underwent elective primary or complex primary THA using the STAR approach, all performed by a senior surgeon. Our primary objectives were to evaluate efficacy by measuring implantation accuracy using postoperative radiographs and safety by recording postoperative complication rates, including dislocation and infection, at a mean follow-up of two years.

## Materials and methods

This single-blinded, mid-term follow-up study was conducted after obtaining approval from the Academic Institutional Scientific Research Board (196/2024). Data were collected from the regional academic arthroplasty registry (ART).

The study involved adult patients (>18 years) with unilateral end-stage hip arthritis who underwent elective primary or complex primary unilateral THA via the STAR approach between May 2019 and December 2023. Complex primary included developmental deformities, dysplasia Hartofylakidis type A and B and post-traumatic or osteotomy arthritis. Patients with severe hip dysplasia, revision surgery, or malignancies were excluded.

Between May 2019 and December 2023, the senior surgeon performed 531 unilateral THAs with the STAR approach using non-offset reamers and broaches. The STAR surgical technique has been previously described in detail [1]. Based on the preoperative spinopelvic classification using standing and sitting lateral views, the surgeon aimed to put the acetabular cup between 40–50^0^ inclination and 15–20^0^ anteversion and the stem in a neutral position [12].

All patients met the eligibility criteria. Nine patients were unavailable for follow-up, mainly because they resided in other countries. At the last follow-up, complete data were available for 522 patients, representing 98.3% of patients. Table 1 displays the demographics and other baseline patients’ data. Most patients included in the study were females, accounting for 63.6% of the total number. The mean age of patients was 65.6 years. Most of them (91.6%) were classified as having ASA scores I and II. Out of all the operations, nearly one-third were primary complex THAs, mainly dysplasia and posttraumatic hip arthritis. The follow-up period they were ranged from three months to five years, with an average of 2.13 years. The mean duration of the operation, measured from the initial incision to wound closure, was 68 min. 91.8% of the patients were discharged by the second postoperative day. Table 2 provides information on surgical intraoperative and radiological data. Most patients (98.5%) underwent uncemented or hybrid THA, with over half receiving uncemented THAs. 81.6% of the patients underwent unconstrained THAs with ceramic on highly crossed-linked polyethylene liner. Dual mobility implants were inserted in almost one-fifth of the THAs in 2B spinopelvic cases. 99% of the femoral stems were collarless. In nearly all cases (94.8%), one or two screws were utilized to secure the cup. A 36 mm femoral head diameter was used in 61.5% of the patients. Table 3 presents other implants’ characteristics.

**Table 1:** The demographics and clinical data of the patients.

Number of patients^*^		522
Age (years)^**^		65.60 (±11.74)
Sex^***^	Female	332 (63.6)
	Male	190 (36.4)
BMI (kg/m^2^)^**^		27.93 (±6.05)
Operated side^***^	Right	290 (55.6)
	Left	232 (44.4)
ASA score^***^	I	139 (26.6)
	II	339 (64.9)
	III	44 (8.4)
Follow-up (years)^**^		2.18 (±1.03)
Preoperative diagnosis^***^	Primary osteoarthritis	420 (80.5)
	Primary complex osteoarthritis	102 (19.5)

^*^The values are given as raw numbers.

^**^The values are given as the mean with the standard deviation (±).

^***^The values are given as raw numbers with the percentages in parentheses.

BMI = body mass index, ASA = American Society of Anesthesiologists score.

**Table 2:** Operative and postoperative radiological data of the patients.

Surgical operation time (min)^*^		68.4 (±11.2)
Length of stay (days)^*^		1.50 (±0.69)
Bearing surface^**^	CoP	456 (87.4)
	MoP	66 (12.6)
Implant fixation^**^	Uncemented	311 (59.6)
	Hybrid	204 (39.1)
	Cemented	7 (1.3)
Acetabular liner^**^	Unconstrained	426 (81.6)
	Dual mobility	94 (18.0)
	Constrained	2 (0.4)
Cup size (mm)^***^		51.5 ± 3.12 (44–62)
Number of acetabular screws^**^	0	27 (5.2)
	1	435 (83.3)
	2	59 (11.3)
	3	1 (0.2)
Femoral head diameter (mm)^**^	36	321 (61.5)
	32	102 (19.5)
	28	59 (11.3)
	22.2	40 (7.7)
Cup orientation^*^	Inclination	42.8 (±4.9)
	Anteversion	19.9 (±8.8)
Stem coronal alignment^**^	Neutral	508 (97.3)
	Varus	14 (2.7)
	Valgus	0 (0)
Stem coronal orientation (degrees)^*^		1.2 (±1.4)

^*^The values are given as the mean with the standard deviation (SD) (±).

^**^The values are given as raw numbers with the percentages in parentheses.

^***^The values are given as the mean with the SD (±) and the range in parentheses.

MoP (Metal on Polyethylene), CoP (Ceramic on Polyethylene).

**Table 3:** Implant type, size and other characteristics.

**FEMORAL STEMs**							
**Stem**		**Conformity**	**Corail**	**Summit**	**Taperloc**	**TwinSys**	**Exeter**			
**N** ^*^		67 (21.5)	183 (58.8)	32 (10.3)	3 (1.0)	26 (8.4)	211 (40.4)			
**Offset** ^*^	**STD**	52 (77.6)	170 (92.9)	30 (93.8)	3 (100)	23 (88.5)	30	1 (0.5)		
	**HO**	15 (22.4)	13 (7.1)	2 (6.2)	0 (0.0)	3 (11.5)	33	5 (2.4)		
							35.5	38 (18)		
							37.5	109 (51.7)		
							44	48 (27.5)		
**Stem size** ^*^	**0**	–	–	–	–	–	118 (55.9)			
	**1**	4 (5.9)	–	3 (10.4)	–	–	55 (26)			
	**2**	9 (13.4)	–	–	–	–	30 (14.2)			
	**3**	10 (14.9)	–	6 (18.7)	–	–	6 (2.8)			
	**4**	11 (16.4)	–	5 (15.6)	–	–	2 (0.9)			
	**5**	18 (26.8)	–	8 (25)	–	–	–			
	**6**	10 (14.9)	–	5 (15.6)	–	–	–			
	**7**	2 (2.9)	–	3 (9.3)	–	–	–			
	**8**	1 (1.5)	8 (4.3)	1 (3.1)	–	–	–			
	**9**	1 (1.5)	9 (4.9)	–	–	4 (15.3)	–			
	**10**	–	22 (12)	–	–	–	–			
	**11**	1 (1.5)	47 (25.6)	–	–	11 (42.3)	–			
	**12**	–	38 (20.7)	–	1 (33.3)	6 (23)	–			
	**13**	–	18 (9.8)	–	–	2 (8.6)	–			
	**14**	–	16 (8.7)	–	–	3 (13)	–			
	**15**	–	18 (9.8)	–	–	–	–			
	**16**	–	5 (2.7)	–	–	–	–			
	**17**	–	–	–	2 (66.7)	–	–			
	**18**	–	2 (1.0)	–	–	–	–			
				**Acetabular cups**						
**Type**	**Pinnacle**	**Trident**	**U motion II plus**	**RM monoblock**	**Exeter X3 Rimfit**	**United dual mobility (UDM)**	**G7 freedom**	**Trilogy**	**Avantage cemented**	
**N** ^*^	214 (41)	207 (39.7)	62 (11.9)	26 (5)	5 (1)	3 (0.6)	2 (0.4)	2 (0.4)	1 (0.2)	
**Size**	**44**	**46**	**48**	**50**	**52**	**54**	**56**	**58**	**60**	**62**
**N** ^*^	4 (0.8)	20 (3.8)	93 (17.8)	101 (19.3)	158 (30.3)	78 (14.9)	37 (7.1)	23 (4.4)	6 (1.1)	2 (0.4)

^*^The values are given as raw numbers with the percentages in parentheses.

STD: standard.

H/O: High Offset.

N: number.

### Perioperative management

All patients were given general anaesthesia. They received intravenous cefuroxime 1.5 g three times and teicoplanin 400 mg twice daily, once before surgery and for 24 h postoperatively. All patients received one gram of tranexamic acid intravenously preoperatively, followed by an 8 mg dose of dexamethasone eight hours postoperatively. No drains were used. Low-molecular-weight heparin was administered for one month postoperatively. Pain management involved 1 g paracetamol and 100 mg tramadol intravenously three times daily and 50 mg of dexketoprofen twice daily for 24–48 h after surgery. Patients were mobilized immediately postoperatively, ambulating with partial weight bearing for 20 days.

### Perioperative evaluation and follow-up

Two independent orthopaedic surgeons who were not involved in the surgical procedures followed up on all patients and conducted the clinical and radiological assessments. The surgeon did not participate in the clinical and radiological assessment. Patients’ demographic, perioperative and postoperative data, including operative time, implant type and sizes, anaesthesia type, length of hospital stay, blood transfusion rate, complication, re-admission and revision rate, were collected. They measured the cup inclination and anteversion, stem alignment and leg length discrepancy (LLD) using the one-month postoperative standardised supine anteroposterior (AP) pelvic X-rays centred over the pubis with the hips in internal rotation (15^0^), including the whole stem length and width.

Cup inclination was evaluated by the angle between the horizontal line connecting the bottom of teardrops and the line indicating the acetabular opening plane [13]. The acetabular anteversion was measured using the method presented by Widmer et al. [14]. The Khalily method was used to assess the femoral stem alignment by measuring the angle between the femoral shaft’s medial endosteal cortex line and the femoral stem long axis in the frontal plane [15]. The Danoff criteria were used to evaluate the safe acetabular zone for implantation [16]. The distance between the lesser trochanters’ tip and the inter-teardrop line was measured to assess the LLD [17].

### Statistical analysis

Standard descriptive methods were utilized for data analysis. The Kolmogorov-Smirnov and Shapiro-Wilk tests verified the normality of the data distribution. Mean and standard deviation or median and interquartile range (IQR) were used to present parametric and non-parametric continuous variables, respectively. Categorical values were presented with absolute and relative frequencies. Statistical analyses were performed using RStudio software (PBC, version 1.4.1103).

## Results

Most cups and stems were impeccably implanted in this cohort, and leg length discrepancy (LLD) was minimal. In the one-month postoperative radiological data, the mean cup inclination was 42.8^0^ (±4.9). The mean cup anteversion was 19.9^0^ (±8.8). 94.1% of the hips fell within the safe zone of inclination; 31 cups were implanted outside, mainly dysplastic hips. According to the Khalily method for evaluating coronal stem alignment, 97.5% of the implants were placed in a neutral position, while only 2.5% were in varus. The mean LLD was 3.3 ± 6.3 mm (Table 2).

The STAR procedure had a minimal complication rate and was considered safe. Of the patients, 58 (11.1%) received a blood transfusion unit during their hospital stay. There were no intraoperative complications, sciatic nerve palsies, thromboembolic events or periprosthetic fractures during the follow-up period. There was a deep infection case in a 63-year-old man that was managed with a two-stage revision. A hip dislocation (0.19%) was recorded in a frail 80-year-old woman who fell from her height during personal hygiene in the bathroom on the first postoperative day. The patient underwent closed reduction and had a hip spica for one month to effectively manage the dislocation. A morbidly obese and two immunosuppressed patients developed a superficial wound infection that was treated with irrigation, debridement, wound care, and oral antibiotics.

## Discussion

Our study reports outcomes of 522 unilateral primary and primary complex THAs performed by a senior surgeon using the STAR approach. This study is the first to report on the STAR approach clinical efficacy and safety in a large cohort. The surgeon achieved excellent surgical precision by successfully inserting the implants with high accuracy. The complication rate of this cohort was minimal, demonstrating an excellent safety profile for this technique.

This study still has some limitations. First, it is a cohort study with no comparison group. Second, the surgeon’s seniority may explain some of the positive outcomes. However, the mean study follow-up is sufficient to explain the approach’s safety and efficacy. Besides, independent and blinded attending surgeons performed follow-up appointments with data analysis, which reduced potential bias.

### Efficacy

**Figure 1:**
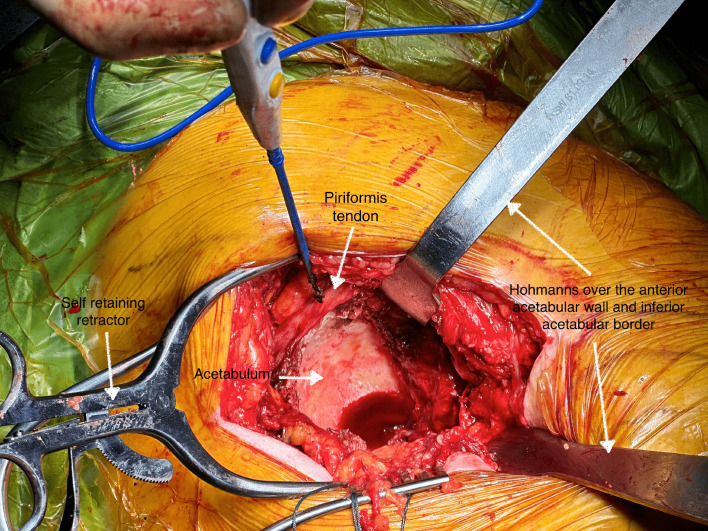
The figure demonstrates the unobstructed acetabular access during the STAR approach. Two self-retaining retractors and two blunt Hohmann retractors are used. The tip of the diathermy and the white arrow show the piriformis tendon.

In our study, we achieved precise and highly reproducible implant orientations and minimal LLD. No revisions were documented for component malposition or LLD. MIS approaches have been linked to higher complication risks due to obstructed access [18]. In anterior or anterolateral approaches, accessing the acetabulum requires radiographic assistance and the challenging lifting of the proximal femur, which may lead to eccentric reaming, stem malposition, and fractures [18]. Using the STAR approach, surgical access to the acetabulum and femur is consistently unobstructed, regardless of patient anatomy, and special instrumentation, traction or fluoroscopy is not required [1]. We firmly believe that this is pivotal to attaining exemplary implant orientation during the implantation. Previous studies supported that PF preservation may hamper the superior acetabular part visualization [2]. The initial tendinous and distal muscular PF part release from the posterior capsule is critical to achieve highly reproducible acetabular access (Fig. 1). This sets the muscle free and allows the PF to move posteriorly and superiorly from the acetabular roof during reaming, following the anterior femoral placement with the retractor placed over the anterior acetabular wall. The PF mobilization can be challenging for dysplastic short-offset hips with long-standing arthritis, possibly due to the loss of muscle elasticity. This reduction in elasticity could cause intraoperative muscle overstretching and damage [19]. During femoral broaching, the PF tendon is typically located away from the intramedullary broaching entry point; the PF tendon attachment is usually found in the greater trochanter medial aspect, anterior and superior to the insertion of obturator internus and gemelli [20]. During femoral broaching, the PF tendon moves further away from the surgical field as the assistant surgeon exerts a longitudinal force on the leg.

### Safety and complications

In our study, we documented limited complications, and the revision rate of our cohort was very low.

#### Neurovascular injuries and blood loss

No cases of sciatic nerve paresis or paralysis were recorded due to consistent identification of the sciatic nerve in all patients operated on with STAR and protection from both the capsule and SER flaps. A higher risk of nerve damage has been reported with other MIS approaches, particularly damage to the lateral femoral cutaneous nerve through the direct anterior approach (DAA) [18]. No vascular injuries were recorded in our series. The STAR approach is well-distanced from major vascular structures. The vessels encountered during surgery are typically small MCFA branches found around the SERs’ tendons, easily identified and safely cauterized [21]. A recent Mayo Clinic cadaveric study demonstrated that DSA, a minimally invasive posterior approach that releases and reattaches the PF tendon, causes less soft tissue damage than DAA, thereby minimizing blood loss [22]. The limited perioperative blood loss and need for transfusion in our series can be mainly attributed to the avoidance of significant vascular structures, the soft-tissue preservation and the shorter incision [1].

#### Wound complications

A minimal wound complication rate was observed using the STAR technique. This may be attributed to the minimal approach invasiveness, the preservation of muscle structures such as piriformis, and the short surgical time [1]. The surgeon’s experience may also explain short duration and low complications. In addition, the STAR surgical incision is exposed to less tension than the greater trochanter may apply to the incisions of the lateral gluteal area, contributing to less stress on the incision edges and, subsequently, better wound healing [1]. During the STAR procedure, the skin incision is made at a distance from the perianal and groin regions, allowing a sterile environment that reduces the contamination risk [1].

#### Dislocations and fractures

No intraoperative or postoperative fractures occurred, with only one dislocation reported in our group. Recent meta-analysis data supports that posterior THA approaches have a similar risk of postoperative hip instability than anterior or lateral approaches [23]. Data from the Dutch Arthroplasty Register, containing over 175,000 primary THAs, reveals that the DSA approach, a mini posterior approach where PF is tenotomised and reattached to the greater trochanter via transosseous channels, poses an equal dislocation risk as DAA and a lower risk than the posterolateral approach [24]. The STAR approach is a posterior MIS approach that offers additional benefits over DSA due to PF retention [1]. Our case series had an extremely low dislocation and zero periprosthetic fracture incidence, which can be attributed to various factors. These include the surgeon’s experience, large femoral heads, the SER and posterior capsule tissue restoration, and the unobstructed acetabular and femoral access that allows optimal implant positioning without stretching the surrounding tissues.

Our initial experience precludes that the STAR approach is easy to perform and offers an excellent acetabular and proximal femoral view using no specialized tools or retractors. It can effectively be used in primary and complex dysplastic hip cases, regardless of implant type or fixation technique [1]. A previous study reported shorter hospital stays, lower transfusion rates, and earlier functional improvement than the DSA approach [1]. However, comparative studies with other standard approaches and the learning curve of the approach have not been evaluated. PF preservation has played a significant role in achieving these positive outcomes. Advancements in understanding the PF muscle role in hip joint stability have led to muscle-preserving hip techniques [1–9]. Recent literature on the posterior approach supported that PF preservation results in significantly less contiguity, atrophy, muscle grade and bulk deterioration, muscle fatty infiltration, and better early functional improvement than reattaching the PF muscle [25]. Several PM-preserving studies alone or with other SHERs [1–9] reported a limited dislocation rate and found short-term benefits in improved walk test, patient satisfaction, blood loss and pain control but similar long-term functional benefits compared to the standard approach [3, 4]. PF preservation for hip hemiarthroplasty may offer functional advantages and a favourable safety profile and has the potential to better maintain pre-injury mobility levels compared to the conventional lateral approach [5]. Table 4 summarises the most important findings of the PF-preserving hip arthroplasty cohort or comparative studies reported. These findings highlight the importance of PM and the necessity for additional research into muscle-preserving methods.

**Table 4:** PM-preserving cohort or comparative studies to standard approaches.

Authors/Year	Study design	Study group	Comparison group	Patients’ No./Surgeries	Outcome
Kenanidis et al. [1]/2023	Comparative cohort	STAR	DSA	400/THAs	STAR had earlier functional improvement, less hospital stay
Siddappa VH et al. [2]/2020	Case series	PM-preserving	No	150/THAs	Less visualisation but no dislocation at six months f.u.
Tan BKL et al. [3]/2020	RCT	PM-preserving	Standard posterior	100/THAs	Same 10-year functional outcomes. PM-sparing improved muscle volume and grade
Khan RJK et al. [4]/2012	RCT	PM-preserving	Standard posterior	100/THAs	Short-term benefits in PM-sparing (better 6-min walk test at 2 weeks and satisfaction at 6 weeks)
Charity J et al. [5]/2023	Retrospective comparative	SPAIRE	Lateral	285/HAs	SPAIRE returned to pre-injury level better than lateral at 3.5 years
Moussallem CD et al. [6]/2012	Case series	PM-preserving	No	226/THAs	No dislocations after 3 years f.u.
Wang T. et al. [7]/2021	Prospective comparative	PM-preserving	Standard posterior	126/THAs	SHER-sparing reduced blood loss, hospital stay, time to mobilisation, stair use and transfusion
Viberg B et al. [8]/2023	Historical comparative cohort	PM-sparing	Standard posterior	527/HAs	PM-sparing showed a 50% reduction in dislocation and reoperation rates.
Apinyankul R et al. [9]/2023	Prospective comparative cohort	PM-sparing	Standard posterior	321/HAs	PM-sparing group had lower dislocation rate, mortality, and higher functional scores

PM: piriformis, THA: total hip arthroplasty, f.u.: follow-up, SHER: short hip external rotators, SPAIRE: Spare Piriformis and Internus, Repair Externus), DSA: Direct Superior Approach, STAR (Superior Transverse Atraumatic Reconstruction), HA: hemiarthroplasty, RCT: randomised control trial, No: number.

## Conclusions

The STAR approach for primary and primary complex THA, retaining PF, is a safe and consistently efficient method that results in excellent early-to-mid-term outcomes. It provides unobstructed access to the acetabulum and proximal femur while preserving the PF and can be easily extended if necessary. The procedure uses standard instrumentation for all patients, implant designs and fixation techniques. Using the STAR approach, the surgeon can expect minimal blood loss, shorter hospital stays, excellent wound healing, and fewer skin complications. Besides retaining the PF, restoring the posterior soft tissue structures, and optimally placing the implants through this approach, along with using large femoral heads, provide a limited risk of hip instability and perioperative fractures.

## Data Availability

All research data is available upon request
